# The Role of Social Novelty in Risk Seeking and Exploratory Behavior: Implications for Addictions

**DOI:** 10.1371/journal.pone.0158947

**Published:** 2016-07-18

**Authors:** Simon Mitchell, Jennifer Gao, Mark Hallett, Valerie Voon

**Affiliations:** 1 Department of Psychiatry, University of Cambridge, Cambridge, United Kingdom; 2 Cambridgeshire and Peterborough NHS Foundation Trust, Cambridge, United Kingdom; 3 Human Motor Control Section, NINDS, National Institutes of Health, Bethesda, Maryland, United States of America; 4 Alpert Medical School of Brown University, Providence, Rhode Island, United States of America; 5 Behavioural and Clinical Neuroscience Institute, University of Cambridge, Cambridge, United Kingdom; Radboud University Medical Centre, NETHERLANDS

## Abstract

Novelty preference or sensation seeking is associated with disorders of addiction and predicts rodent compulsive drug use and adolescent binge drinking in humans. Novelty has also been shown to influence choice in the context of uncertainty and reward processing. Here we introduce a novel or familiar neutral face stimuli and investigate its influence on risk-taking choices in healthy volunteers. We focus on behavioural outcomes and imaging correlates to the prime that might predict risk seeking. We hypothesized that subjects would be more risk seeking following a novel relative to familiar stimulus. We adapted a risk-taking task involving acceptance or rejection of a 50:50 choice of gain or loss that was preceded by a familiar (pre-test familiarization) or novel face prime. Neutral expression faces of males and females were used as primes. Twenty-four subjects were first tested behaviourally and then 18 scanned using a different variant of the same task under functional MRI. We show enhanced risk taking to both gain and loss anticipation following novel relative to familiar images and particularly for the low gain condition. Greater risk taking behaviour and self-reported exploratory behaviours was predicted by greater right ventral putaminal activity to novel versus familiar contexts. Social novelty appears to have a contextually enhancing effect on augmenting risky choices possibly mediated via ventral putaminal dopaminergic activity. Our findings link the observation that novelty preference and sensation seeking are important traits predicting the initiation and maintenance of risky behaviours, including substance and behavioural addictions.

## Introduction

The presence of novelty appears to influence risk taking. Novel financial products with poorly understood risks have been implicated as one of the main factors behind the recent financial crisis[1]. Similarly, the proliferation of novel betting products suggests that exposing gamblers to novel risk situations may be a profitable way to encourage risk-taking[2]. Sexual risk-taking, alcohol and drug use is enhanced in young travelers on holiday [3] [4], potentially suggesting that novel environments play a significant role in their behaviour. This influence of novelty on risk taking has implications for disorders of pathology such as addictions. The trait of sensation seeking has also been shown to predict subsequent adolescent binge drinking and externalizing behaviours [5,6]. Here we focus on the influence of social novelty priming on risk taking in healthy volunteers.

Novelty has also been shown to influence choice in the context of uncertainty and reward processing. The preference for the novel stimulus in an uncertain context is associated with activation of the ventral striatum and midbrain [7]. A novel context also enhances reward processing in conditions of a low probability of reward [8]. In that study, novel and familiar images were paired with conditioned reward-predictive cues of differing probabilities (P = 0, 0.4, 0.8). Under the low reward probability (P = 0.4) condition, novel images enhanced striatal reward activity and contextual novelty was associated with enhanced hippocampal activity. Cues predicting novel images elicited significantly higher substantia nigral activation than cues predicting familiar stimuli. The authors suggest that the findings support the model of a hippocampal-dopaminergic ventral tegmental area functional loop playing a significant role in detection of novelty and its subsequent enhanced long term potentiation and learning [9] leading to increased striatal reward processing.

This present study investigates the influence of novel or familiar contextual primes on judgements of risk in healthy volunteers. Rather than a focus on novelty preference in an uncertain context [7], the present study focuses on the influence of a novel context on priming risk taking choices. Subjects were tested behaviourally and a subset were scanned using functional MRI while performing a different version of the same task. We adapted a risk-taking task involving acceptance or rejection of a 50:50 choice of gain or loss. In healthy volunteers scanned with this risk task, regions encoding subjective value have been shown to increase in activity to potential gains and decrease in activity to potential losses during risk taking [10]. Here we introduce a novel or familiar prime preceding the risk choice and focus on imaging correlates to the prime that might predict risk seeking in healthy volunteers. We hypothesize that subjects would be more risk seeking following a novel relative to familiar prime. We further hypothesize that greater risk seeking across subjects would correlate with greater ventral striatal and hippocampal activity to the novel versus familiar prime.

## Materials and Methods

### Behavioral Study

Written informed consent was obtained from all participants, and the study was conducted in accordance with the guidelines of the National Institutes of Health Institutional Review Board (IRB) committee. This study was approved by the NIH IRB.

Healthy volunteers were recruited from the National Institute of Health healthy volunteer database. Subjects were included if they were 18 years of age or older, had no major psychiatric or medical illnesses and were medication free. Subjects were screened for psychiatric disorders using the Mini International Neuropsychiatric Inventory [11].

### Task

#### Familiarization and practice

In the behavioural familiarization phase, subjects were randomly shown one of six neutral-expression faces (three male, three female from The Karolinska Directed Emotional Faces database [12]) and asked to press “F” with their left index finger if the face shown was female, and to not respond if the face was male. Each face was shown for 1200 ms followed by a white screen jitter of 250–750 ms. Each face was shown 17 times over a 3 minute period. These facial stimuli were considered Familiar.

**Fig 1:**
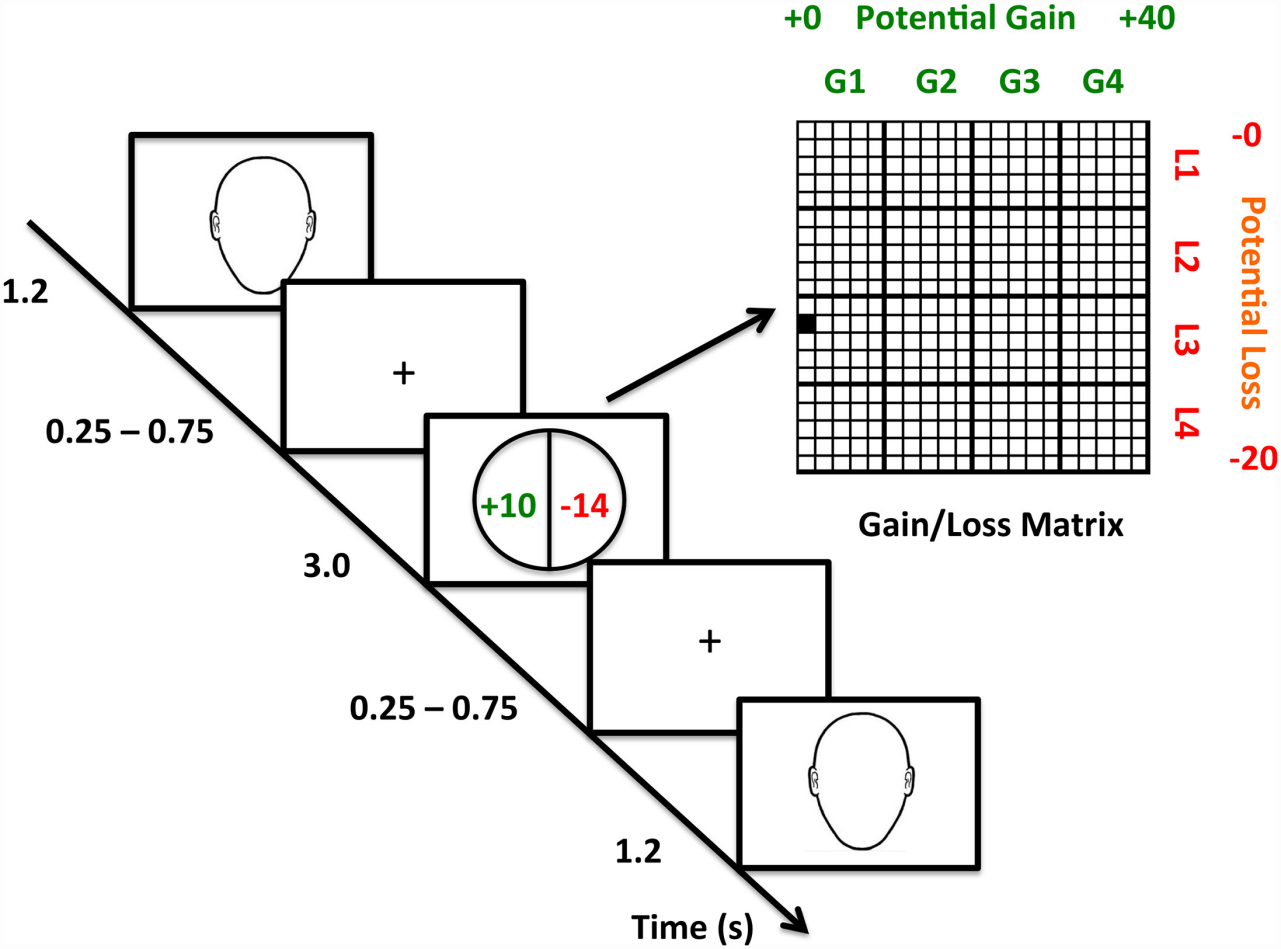
Novelty prime risk task. Subjects were first familiarized to neutral faces (not shown). Subjects identified if the prime was male or female (left index finger for female) and chose to reject (right index finger) or accept (right middle) the 50:50 gamble. All possible gain amounts varying from 0 to +40 (green) were paired with all possible loss amounts varying from 0 to -20 (red).

In the practice task subjects were randomly shown one of the same six Familiar faces as in the familiarization session. They were asked to press “F” with their left index finger if the face shown was female and not respond if the face was male. Each face was followed by a risk circle[10] consisting of a circle with a vertical line running down the middle dividing it into two equal halves (Fig 1). On the left side of the circle was the gain amount shown in green with a + sign (e.g.+ $20). On the right side of the circle was the loss amount shown in red with a—sign (e.g.- $20). Similar to the original task design, the gain amounts varied from +10 to +40 in +2 increments and were randomly paired with the loss amounts, which varied from -5 to -20 in -1 increments (The relationship between gain and loss amounts are shown in Fig 1).

Subjects were told they had a 50% chance of either winning the amount of money shown on the left in Green or losing the amount of money shown on the right in Red. Based on the 50/50 odds, subjects were asked to press “J” with their right index finger if they strongly rejected the gamble, “K” with their right middle finger if they weakly rejected it, “L” with their right ring finger if they weakly accepted it, and “;” with their right pinky if they strongly accepted the gamble. Above each gamble circle, “strongly reject → strongly accept” was displayed to remind the subjects which keys related to their choices. Subjects were told to make their selection as quickly as possible once the risk circle appeared, to move on if they made a mistake and not to try and correct that mistake by pressing extra keys. They were instructed that the goal of this task was to make as much money as possible. The computer calculated the amount of money they were winning based on their choices, to a maximum of $30. Each cycle lasted 5200 ms and consisted of a familiar face being shown for 1200 ms, followed by a jitter between 250 to 750 ms, the risk circle being shown for 3000 ms, and then another jitter between 250–750 ms. The practice session was designed to allow subjects to familiarize themselves with the risk circle and the process of accepting or rejecting the gamble. They were told that this session was for practice only and would not count toward their final winnings. The session lasted 7 minutes, cycling each familiar face a further 13 times. Thus a total of 30 repetitions were completed between the familiarization and risk practice sessions. Written and verbal instructions were provided for each session.

#### Test

Subjects were given the same instructions as in the risk practice session apart from being informed that their choices would count toward their final winnings. This session used fourteen faces with 6 Familiar faces and eight Novel neutral-expression faces that the subjects had never seen before (four male, four female). Each cycle consisted of a randomly selected face, a jitter, a risk circle and another jitter, with timings and gain and loss amounts identical to the practice session. A total of 512 trials (256 each for the non-novel and novel faces) were carried out to cover all permutations of gain and loss combinations and novel versus familiar faces. Subjects underwent three sessions of 171 trials with each session lasting 15 minutes. Approximately 50%-60% of the faces were novel. Subjects were given a maximum of 10 minutes rest between each session.

They were further informed that the computer would remember each choice and randomly select a choice to play and that they would be compensated a proportion according to their choice. Each experiment was run on a Dell Laptop running Windows XP. The experiment was programmed using E-Prime.

The primary outcome measure was the percent of risky choices. Secondary outcome measures included response times to both gender decisions and risk choices.

Upon completion of the risk session, subjects were asked to complete the Temperament and Character Inventory (TCI) Novelty Seeking subsection[13].

### fMRI Study

#### Familiarization

The same familiarization phase was conducted outside of the fMRI scanner. Subjects were randomly shown one of the same six neutral expression non-novel faces (three male, three female) used during behavioral familiarization phase. As in the behavioral study, they were asked to again immediately press “F” with their left index finger if the face shown was female, and to not respond if it was male.

This was followed by a practice task. Subjects were shown one of the same six neutral expression familiar faces and asked to immediately press “F” with their left index finger if the face shown was female and to do nothing if the face was male. Subjects were then shown the risk circle and asked to press “J” with their right index finger if they fully rejected the gamble and “K” with their right middle finger if they fully accepted the gamble. This is in contrast to the behavioral task, where four options had been available. Subjects were asked to make their selection as quickly as possible and not to try to correct any mistakes. This session lasted 5 minutes.

#### Test

Subjects saw a randomly selected novel or familiar face for 1200 ms, a jitter between 250–750 ms, the risk circle for 3000 ms, and another jitter between 250–750 ms prior to the next trial (Fig 1). The same gain and loss amounts were used as in the behavioral session. Subjects were tested in three sessions with 120 risks per session and 60% novel faces. To identify the female prime, subjects used their left index finger to press on a response box. To choose to reject or accept the gamble, subjects used their right index or middle finger respectively to press on a different two-button response box. Subjects were told that this session would count towards their final winnings.

Imaging was performed using a 1.5T GE MRI Scanner. We acquired T2 weighted echoplanar images (EPI) [slice thickness, 2 mm; 30 slices; repetition time (TR), 2.1 s; echo time (TE), 33 ms, flip angle, 90°; voxel size 3x3x3 mm; matrix, 64x64; field of view (FOV), 24 mm]. Data processing was conducted using SPM5 (http://www.fil.ion.ucl.ac.uk/spm/software/spm5/) with standard preprocessing: slice timing, realignment, normalization to the standard MNI template, and smoothing.

### Data analyses

The data were examined for outliers (>3 SD from group mean) and normality using Kruskal-Wallis test. For the behavioural data, primary analyses examined their percent of risky choice as a function of Valence (Reward, Loss), Magnitude (the magnitudes were combined into 4 levels: Gain:+0 to +40 divided by +10; Loss: -0 to -20 divided by -5), and Novelty (Novel, Familiar). As the percent of risky choices were not normally distributed, we first examined differences across Novel and Familiar using Related Samples Friedman’s two-way ANOVA by Ranks comparing percent risky choices for Novel Gain (average of the percent risky choices for each individual across the 4 Gain levels to Novel primes), Familiar Gain (average for each individual across the 4 Gain levels to Familiar primes), Novel Loss and Novel Familiar. As this was significant, we then compared Novel Gain versus Familiar Gain and Novel Loss versus Familiar Loss separately using Related-samples Wilcoxon Signed Rank Test. We then examined the effects of Magnitude using Related Samples Friedman’s two-way ANOVA by Ranks to compare percent risky choices for the 4 different magnitude levels for Gain and for Loss. As this was significant, we then conducted post-hoc comparisons compared the effects of Novel versus Familiar for each Magnitude separately for each Valence (e.g. Gain +0 to +10 Novel versus Gain +0 to +10 Familiar; Loss -10 to -15 Novel versus Loss -10 to -15 Familiar). We examined reaction time to the priming stimuli as a function of choice preference using a repeated measures ANOVA with within-subject factors of Novelty (novel versus familiar) and Risk (accept or reject). We also compared the reaction time during the risk decision as a function of exposure to a novel versus familiar prime using a paired t-test.

The EPI data were preprocessed using SPM8 including slice timing, realignment and unwarping, normalization and smoothing. For the imaging analyses, we focused on our *a priori* hypothesis of the influence of priming on choice preference but also modeled the priming, risk choice and jitter phases. We assessed the contrast of the novel versus familiar prime and used a regression analysis at the second level to assess the relationship with the percentage of risky choices for novel versus familiar primes (i.e. the average percentage risky choices for novel—average percentage risky choices for familiar for each individual).

Whole brain voxel-wise group comparisons were performed using a cluster extent threshold correction. The cluster extent threshold correction for a voxel size of 3x3x3, smoothing 11.5 mm FWHM was calculated at 16 voxels at P < 0.001 whole brain uncorrected, which corrected for multiple comparisons at P < 0.05 assuming an individual-voxel Type I error of P = 0.01 [14]. We further conducted exploratory analyses focusing on the sub-factors of novelty seeking score, particularly the sub-factor of exploration, from the TCI. As we identified putaminal activity correlated with risk taking behavior, we focused specifically on the relationship with putaminal activity.

## Results

### Behaviour

Twenty-four healthy volunteers (12 male, 12 female; mean age 43 years old, SD 15.1 years) were recruited for the behavioral study. Eighteen of the first twenty-four behavioral subjects tested were recruited for the fMRI study (10 male, 8 female; mean age 44 years old, SD 13.5 years).

There was a main effect of the Novel versus Familiar prime on the percent risky choices (Related Samples Friedman’s two-way ANOVA by Ranks p = 0.025). Novel primes increased the percent risky choices versus Familiar primes in both the Gain (Related-samples Wilcoxon Signed Rank Test, p = 0.037) and Loss (p = 0.016) conditions. Examining the effects of Novel versus Familiar prime on Gain and Loss magnitude showed differences in the percent risky choices (Related Samples Friedman’s two-way ANOVA by Ranks p<0.0001). On post-hoc analyses, Novel primes increased the percent risky choices versus Familiar primes in the three lowest Gain and intermediate Loss magnitudes (Related-samples Wilcoxon Signed Rank Test, Gain +0 to +10, p = 0.001; Gain +10 to +20, p = 0.028, Gain +20 to +30, p = 0.033, Gain +30 to +40, p = 0.836; Loss -0 to -5, p = 0.094; Loss -5 to -10, p = 0.110; Loss -10 to -15, p = 0.007; Loss -15 to -20, p = 0.424). Assuming a highly conservative Bonferroni threshold for the post-hoc analyses, the lowest Gain magnitude remained significant after correction for multiple comparisons (Bonferroni corrected, p<0.006).

**Fig 2:**
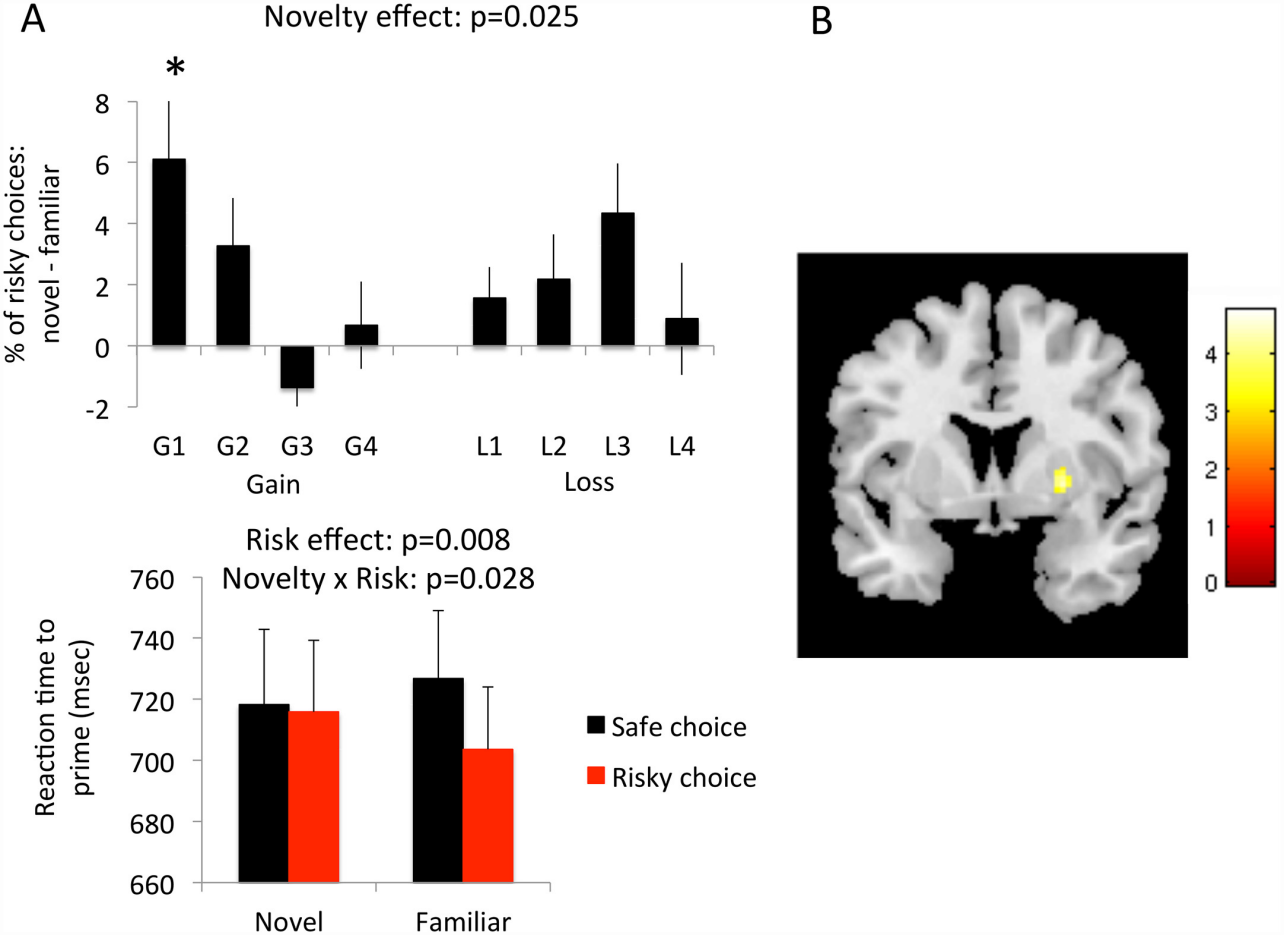
Behavioural and imaging findings. A. The top graph shows the percentage of risky choices of novel versus familiar primes as a function of different gain (+0 to +40 divided by +10) and loss magnitudes (-0 to -20 divided by -5). The bottom graph shows the reaction time to the novel and familiar prime as a function of the safe or risky choice. *post-hoc p = 0.001 significant after Bonferroni correction. B. The coronal image shows that greater right putaminal BOLD activity of the novel versus familiar prime contrast was positively correlated with greater subsequent risky choices across subjects following exposure to the novel versus familiar prime.

We examined the effects of reaction time to the priming stimuli as a function of accepting or rejecting the risky choice using a mixed measures ANOVA with within-subject factors of Novelty (novel versus familiar) and Risk (accepting or rejecting). We show no main effect of reaction time on Novelty (F(1,23) = 0.192, p = 0.665), with a main effect of Risk with faster reaction times to the prime when accepting rather than rejecting the risky choice (F(1,23) = 8.400, p = 0.008) (Fig 2). There was a Novelty x Risk interaction (F(1,23) = 5.469, p = 0.028) in which subjects had faster reaction times to familiar primes when they subsequently accepted versus rejected the risk (mean difference 23.101 (95%CI: 9.311–36.892), p = 0.002) with no difference to novel primes (p = 0.687). Subjects also had faster reaction times to familiar compared to novel primes when accepting a risk (12.17 (95%CI: 0.43–23.91), p = 0.043) with no differences when rejecting a risk (p = 0.193).

We compared the reaction time during the risk decision as a function of exposure to a novel versus familiar prime using a paired t-test. In response to novel compared to familiar primes, subjects were slower when making the risk decision (Novel risk choice: 1250. 18 (SD 336.57); Familiar risk choice: 1180.70 (SD 372.70), df = 23, t = 2.213, p = 0.037).

There was no correlation between novelty seeking as measured using the TCI and risk taking in this task (p>0.05).

### Imaging

In the contrast of novel versus familiar primes, there was greater bilateral hippocampal activity (Left: peak voxel in Montreal Neurological Institute coordinates x y z (in mm): -28–24–18, Cluster size = 310, Z = 3.54; Right: 38–20–10, Cluster size = 62, Z = 3.21).

We show that right putamen activity to novel versus familiar primes was positively correlated with risk taking choices (peak voxel in Montreal Neurological Institute coordinates x y z (in mm): 27–2 2; Cluster size = 35, Z = 3.91)(Fig 2). We did not show an influence on hippocampal activity. We then asked if any factors within the novelty seeking factor of the TCI were correlated with putaminal activity to novel versus familiar primes. Bilateral putaminal activity was correlated with the exploratory factor (Right: 32–2 6, Cluster size = 237, Z = 3.38; Left: -28 8–2, Cluster size = 292, Z = 3.22) but not with extravagance, disorganization or impulsivity.

## Discussion

We show that exposure to novel as compared to familiar primes are associated with enhanced risk taking to both gain and loss anticipation in healthy volunteers. Using a highly conservative threshold, this finding was relevant particularly for lowest magnitude of gain anticipation in the context of risk. Greater right putaminal activity in the contrast of novel versus familiar primes also was correlated with greater subsequent risk taking choices and with self-reported exploratory behaviours. Our findings suggest a predictive relationship given that the primes always preceeded, and were temporally dissociated from, risk-taking choices. Furthermore, faster reaction times particularly to familiar compared to novel primes were associated with enhanced risk seeking behaviours. Overall we show that exposure to a novel context can enhance risk seeking behaviours to risky small gains. This effect was predicted by faster reaction times to familiar contexts and greater right putaminal activity to novel versus familiar contexts.

Novelty has been suggested to have a contextually enhancing effect on reward-processing [8,15]. In a study comparing novel and familiar images paired with reward predicting cues, novelty had the greatest influence on striatal reward probability in the low reward probability condition (P = 0.4) as compared to no (P = 0) or high (P = 0.8) reward probabilities. Our current study focused on the influence of novel and familiar contexts on a fixed risk preference at the highest risk with P = 0.5 of winning or losing differing magnitudes. Thus, the influence of novelty in the reward cue prediction paradigm, rather than a role for low probability, may be influenced by the degree of uncertainty since P = 0.4 has greater uncertainty and variance in outcome relative to P = 0 or P = 0.8. However, as we did not modify risk probability in our current task, we are not able to address the role of a differential effect on risk probability. That a novel context was associated with prolonged reaction time during the assessment of risk as compared to a familiar context further suggests that novelty does not simply result in rapid impulsive poorly-considered risky decisions. There was no evidence of a novelty-mediated enhancement in motivational effects as subjects were slower to make a decision during the choice phase when exposed to novel versus familiar contexts.

We show that novelty has the greatest effect in the lowest gain magnitude condition suggesting an influence on the anticipation of the gain outcome rather than a general influence on the processing of risk or probability. The processes of novelty and reward anticipation in the context of risk may be linked by dopaminergic function. In primate and human studies, novel stimuli are associated with an increase in phasic dopaminergic activity [16]; [17]; [18]. Absolute novelty stimulus is associated with blood oxygen level dependent (BOLD) activity in the substantia nigra and ventral tegmental area in functional MRI (fMRI) studies in humans [16]. An enhancement in phasic dopaminergic ventral tegmental and hippocampal activity to a novel priming context may influence the subsequent processing of reward anticipation in the context of risk. Functional MRI studies of this risk task in healthy volunteers show that the anticipation of reward in this risk task is associated with increasing activity in regions predicting reward such as ventromedial prefrontal cortex and ventral striatum[10] with lower magnitudes associated with lower activity and higher magnitudes associated with higher activity. The influence of novelty on gain magnitude expectation may be related to a ceiling effect. An increase in activity to a novel context (e.g. enhanced dopaminergic release or striatal activation) may have more of a priming effect on the expectation of a low gain associated with lower activation (e.g. of dopamine release or striatal activation) relative to expecting a higher gain associated with greater activations. A novel context may act as a prime to enhance the gain or proportion of dopaminergic neurons firing spontaneously and thus capable of phasic firing to reward hence increasing neuronal activity during reward anticipation [19,20]. Alternatively, preclinical studies suggest that novelty may be treated similarly to a rewarding stimulus[21], with rats demonstrating reduced self-administration of amphetamine during exploration of novel stimuli [22] and place preference for environments with novel stimuli[23]. Thus, a novel context may itself act as an incentive augmenting the prediction of low reward magnitudes and may have greater influence on a risky unfamiliar context[15].

Our findings also suggest a role for inter-individual differences in risk taking propensity. Greater putaminal activity to novel versus familiar contexts and faster reaction times to familiar contexts predict greater risk taking behaviours. This may be related to an enhanced activation to novelty or enhanced habituation to familiar contexts reflected in greater putaminal activation to the novel versus familiar contrast along with enhanced reaction times during the decision of whether the subject was male or female. Based on the model of a hippocampal-dopaminergic ventral tegmental area functional loop [9], we may have predicted ventral striatal rather than putaminal activation. Proactive motor regulation may also influence decision making in the context of risk which may also explain our findings of putaminal activation[24]. This study suggests that the executive processes that govern motor control and those involved in complex decision making, such as evaluating monetary risk, may have closely connected mechanisms.

The results highlight the influence of novel social contexts on enhancing risk taking behaviours, possibly by enhancing the anticipation of low gain magnitude. Our findings may link the observation that novelty preference and sensation seeking are important traits predicting the initiation and maintenance of risky behaviours, including substance and behavioural addictions [25,26] [5,6].
